# Hoarseness of voice: An alarming sign to recheck the position of naso-gastric tube

**DOI:** 10.4103/0019-5049.72666

**Published:** 2010

**Authors:** Pratibha Jain Shah, KP Dubey

**Affiliations:** Pt. J.N.M. Medical College and Dr. BRAM Hospital, Raipur, (C.G.), India

Sir,

Naso-gastric tube (NGT) is usually indicated for enteral feeding or aspiration of intestinal secretions or stomach wash in cases of suspected poisoning. But before using this tube for any procedure, it is imperative to check and confirm the correct position of the distal end of the tube. Because, occasionally the tube may inadvertently enter the airway instead of the gastrointestinal tract.[1] One can easily imagine the unfavorable outcome (from aspiration pneumonitis, pneumothorax, collapse of alveoli to even death), once the tube remained undiscovered in trachea and enteral feeding or stomach wash is started.[2,3] We report two patients scheduled for emergency exploratory laprotomy having acute onset hoarseness of voice.

A 28-year-old male with perforation peritonitis and a 40-year-old female with intestinal obstruction were scheduled for emergency exploratory laprotomy, with NGT (16 no size) *in situ*. While doing preoperative assessment, hoarseness of voice was noticed in both the cases. It was found to be acute in onset. As we could not get any other reason other than possibility of NGT in trachea, NGT was removed without confirmation of position in first case. As soon as the tube was taken out, patient regained normal voice. With previous experience, it was planned to confirm position of NGT with the help of direct laryngoscopy in second case. After explaining the procedure to the patient, direct laryngoscopy was done and it was found that the tube was entering into the larynx instead of oesophagus. The tube was taken out following confirmation of wrong positioning. NGT was repositioned in oesophagus under direct laryngoscopy and guiding with the help of Magill’s forcep after intubation, in both the cases.

**Figure 1:**
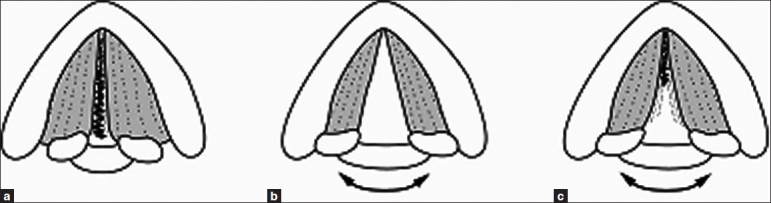
Axial view from above the vocal folds. (a) complete vocal cord closure upto cartilagenous portion. (b and c) During phonation- abduction and adduction of vocal cords. While the membranous portion of vocal cords vibrate, cartilagenous portion remains open and allow constant flow of air during phonation

Hoarseness is usually caused by a problem in the vocal cords. During phonation, the vocal cords meet in midline and as air leave the lungs; they vibrate producing sound [Figure 1]. Anything (FB, tumour, polyp, inflammatory reaction, vocal cord palsy), which prevents proper approximation of cords, leads to difficulty in producing sound when trying to speak or change in pitch or quality of voice. Voice may sound weak, scratchy or husky. Possible common causes of sudden hoarseness of voice can be: Tonsillitis, Adenoiditis, Heavy smoking, Alcoholism, Excessive crying, Singers, Irritant gas inhalation, Viral illness, Ingestion of caustic liquid, Foreign body, Allergies.

Although awake, healthy persons have protective airway reflexes that prevent entry of any FB into the trachea, but general debility and weakness lead to partial suppression of the laryngeal reflexes.[2] Galley states, “It is well known that even in the conscious patient the larynx has a greater tolerance for foreign bodies which do not move”.[4] Respiratory distress, coughing, straining, and retching are not normal reactions to the passage of a Ryle’s tube. When these occur to excess in any patient, particularly in the exhausted, toxic, or otherwise debilitated, the tube should be under suspicion until proved, by positive gastric aspiration, to be in the stomach.[2] So one must always be suspicious about the incorrect position of the Ryle’s tube.

There are various methods to ascertain the position of the tube in the stomach, like aspirating 2 mls of stomach content with a syringe and pour on litmus paper (turn blue litmus paper red), injecting 5 ml of air into the tube (whooshing sound over the epigastrium with stethoscope), X-ray chest and upper abdomen, pH testing of feeding tube aspirates, capnograph, calorimetric CO_2_ detector.[5] Although radiologic confirmation of tube placement remains the “gold standard”, there is growing evidence that pH testing of feeding-tube aspirates can reduce (although not totally eliminate) reliance on X-rays used for this purpose.[5]

We, therefore, recommend that any case of Naso-gastric tube *in situ* having acute onset hoarseness of voice, one must reconfirm exact position of Naso-gastric tube, with any of the above mentioned technique before starting procedure to avoid fatal consequences.
